# Moderate neonatal hypoglycemia and adverse neurological development at 2–6 years of age

**DOI:** 10.1007/s10654-018-0425-5

**Published:** 2018-07-20

**Authors:** Ronny Wickström, Beatrice Skiöld, Gunnar Petersson, Olof Stephansson, Maria Altman

**Affiliations:** 1 Department of Women’s and Children’s Health, Karolinska Institutet, Stockholm, Sweden; 2 Department of Medicine Solna, Clinical Epidemiology Unit, Karolinska Institutet, Stockholm, Sweden; 3 Humanitas University, Rozzano, Milan, Italy

**Keywords:** Newborn, Blood glucose, Outcome, Developmental delay

## Abstract

To determine whether moderate neonatal hypoglycemia in otherwise healthy infants is associated with adverse neurodevelopmental outcome in pre-school children. Population-based cohort study with prospectively collected register data from Sweden. All singletons born July 1st 2008 through December 31st 2012 (n = 101,060) in the region were included. Infants with congenital malformations, infants treated in neonatal intensive care unit, infants with inborn errors of metabolism and infants to mothers with diabetes were excluded. Infants were followed-up until 2014, at 2–6 years of age. Exposure was neonatal moderate hypoglycemia. Main outcomes were a compiled neurological or neurodevelopmental outcome; any developmental delay; motor developmental delay; and cognitive developmental delay. In adjusted regression analyses, the odds ratio (OR) of any neurological or neurodevelopmental outcome was 1.48 (95% confidence interval: 1.17–1.88) in hypoglycemic compared to normoglycemic infants. The adjusted risk of any developmental delay was more than doubled (OR 2.53 [1.71–3.73]), the adjusted risk of motor developmental delay was almost doubled (OR: 1.91 [1.06–3.44]) and the adjusted risk of cognitive developmental delay was almost tripled (OR 2.85 [1.70–4.76]). Infants with early neonatal hypoglycemia (< 6 h) had a double risk (OR 1.94 [1.30–2.89]) of any neurological or neurodevelopmental outcome and a tripled risk of cognitive developmental delay (OR 3.17 [1.35–7.43]), compared to normoglycemic infants. In the first population-based study on this topic, we show that moderate neonatal hypoglycemia is associated with increased risks of impaired neurodevelopment. Current treatment routines for uncomplicated hypoglycemia should be followed.

## Introduction

Hypoglycemic episodes requiring treatment during the first days of life are common (5–15%) [1]. Whereas it has long been known that prolonged, symptomatic or severe hypoglycemia can cause damage to the newborn central nervous system [2–6], the effects of transient or moderate hypoglycemia in low-risk infants has been a subject of controversy [7, 8]. Some argue that transient neonatal hypoglycemia should be regarded as a harmless, physiological process while others see it as a potential threat to the newborn brain.

The general assumption is that asymptomatic infants with transient hypoglycemia are at very low risk of neurologic complications [9, 10] and this is supported by some studies [11, 12]. However, there are indications that even moderate neonatal hypoglycemia may be associated with structural brain abnormalities [3, 4], impaired neurodevelopment [5], impaired executive function and visual motor function [7] and poor school performances [13]. Previous studies on transient or moderate hypoglycemia have been either small, performed on selected populations, or without data on important confounders. There have been several calls for better scientific evidence on this topic [1, 13–15].

There is a lack of studies of healthy, term and near-term infants with moderate hypoglycemia, without need for intravenous glucose fluids or admission to the neonatal intensive care unit [8, 15]. We performed a large population-based study of moderate neonatal hypoglycemia and its correlation to neurodevelopmental diagnoses in children up to 6 years of age.

## Methods

### Study design and data sources

We performed a population-based cohort study with prospectively collected data. Information on mother, pregnancy, delivery and infant was obtained from computerized antenatal, obstetrical and neonatal electronic medical records including births in the counties of Stockholm and Gotland, Sweden (the Stockholm-Gotland Obstetric Cohort [16]). Within this region, the same medical record system (Obstetrix, Cerner Inc.) is used for all antenatal, delivery and postnatal care units. Clinical input is transferred to a database on a daily basis, and detailed information on maternal, pregnancy, delivery and infant health parameters is collected from 2008 and onwards.

Via the individual Swedish National Registration Number assigned to each Swedish resident at birth or immigration [17], data from the Stockholm-Gotland Obstetric Cohort was linked to two population based registries held by the Swedish National Board of Health and Welfare. The Swedish National Patient Register provides ICD-10-codes (International Classification of Diseases 10) of primary and secondary diagnoses at discharge for all patients admitted to hospital care since 1987 and from specialized outpatient care units since 2001. The Cause of Death Register collects data on date and cause of death on all Swedish residents since 1963.

The study was approved by the regional ethics board in Stockholm, Sweden (2009/275-31 and 2012/365-32). Patient information was retrieved from a computerized medical record system and national health registries and there was no informed consent prior to inclusion in the study.

### Study population

From July 1st 2008 through December 31st 2012, 112 256 singleton infants (twins, multiplets and all infants with a malformation diagnosis [ICD-10: Q00-Q99] excluded) were born in the Stockholm-Gotland county. The purpose of this study was to examine otherwise healthy infants and therefore, all infants admitted to a neonatal care unit (n = 9 852) were excluded before analyses. We then excluded infants (n = 170) who received one or more of the following ICD-10 diagnoses of inborn errors of metabolism before end of follow-up: E03; E25; E53.8; E70; E71; E72; E74; E75; E76; E77; E78; and E79. Information on maternal pre-gestational diabetes was collected at the first attendance to antenatal care, generally at 8–12 gestational weeks. Gestational diabetes was screened for by blood glucose test at the antenatal booking and at gestational weeks 25, 29, 32/33 and 37/38, respectively. Infants whose mothers had pre-gestational (n = 484) or gestational diabetes (n = 690) were then excluded because maternal diabetes was considered a possible mediator on the association between neonatal hypoglycemia and neurological outcome. After these operations, 101 060 infants were included in the study cohort.

### Exposure

Exposure was defined as having a diagnosis of transitory neonatal hypoglycemia (blood glucose < 40 mg/dL [< 2.2 mmol/L; to convert from mg/dL to mmol/L, use conversion factor 0.0555]; ICD-10: P70.4; P70.4A; and/or P70.4B) at discharge from the postnatal ward. Blood glucose was measured by bed-side analyses. The unexposed group consisted of all other infants discharged from the postnatal ward. Exposure was categorized into hypoglycemia within the first 6 h after birth (ICD-10: P70.4A); hypoglycemia after the first 6 h after birth (ICD-10: P70.4B); and any neonatal hypoglycemia (ICD-10: P70.4; P70.4A; P70.4B). Iatrogenic neonatal hypoglycemia (ICD-10: P70.3) was not included in the exposure group. Among included high-risk infants (large or small for gestational age infants, and preterm infants), extra feeding was initiated within 1 h after birth and blood glucose was routinely measured just before the newborn’s second meal or before 4 h of age. Blood glucose was then followed-up until there were two consecutive normal values over 47 mg/dL and additional feedings provided when necessary. In low-risk infants, blood glucose was measured only if symptoms of hypoglycemia were present. Infants in need of intravenous glucose infusions were admitted to a neonatal care unit and thereby excluded from the study cohort.

### Outcome

All infants were followed-up until end of 2014, by the time they were 2–6 years of age. Outcome was defined by pre-specified neurologic and developmental diagnoses in the Swedish National Patient Registry or the Swedish Cause of Death Registry. Main outcome was defined as one or more of the pre-specified diagnoses. Secondary outcome was defined as any developmental delay; motor developmental delay; cognitive developmental delay; autism spectrum disorders and attention-deficit/hyperactivity syndromes; tics and stereotypic disorders; and/or epilepsy or febrile seizures. Detailed definitions with ICD-10 codes of the main and secondary outcomes are presented in Table 1.

**Table 1:** Definitions and grouping of neurodevelopmental outcomes

Outcome group	ICD-10 codes
Any neurological or neurodevelopmental outcome (one or more of the following diagnoses)	
Intellectual disabilities	F70, F71, F72, F73, F78, F79
Specific developmental disorders	F80, F81, F82, F83
Autism spectrum disorders	F84
Disorders of psychological development	F88
Attention-deficit/hyperactivity disorders	F90
Tics, stereotypic behavior, stuttering	F95, F98.4, F98.5
Myoclonus, epilepsy and recurrent seizures, status epilepticus	G25.3, G40, G41
Abnormalities of gait and movement, other lack of coordination	R26, R27
Dyslexia and alexia, other symbolic dysfunctions	R48.0, R48.8
Seizures including febrile seizures	R56
**Any developmental delay**	F70, F71, F72, F73, F78, F79 F80.0, F80.1, F80.2, F80.8, F80.9, F81.0, F81.1, F81.2, F81.3, F81.8, F81.9, F82, F83, R26, R27 R48.0, R48.8
**Motor developmental delay**	F82, R26, R27
**Cognitive developmental delay**	F70, F71, F72, F73, F78, F79, F80.0, F80.1, F80.2, F80.8, F80.9, F81.0, F81.1, F81.2, F81.3, F81.8, F81.9, F83, R48.0, R48.8
**Autism spectrum disorders and attention deficit syndromes**	F84.0, F84.1, F84.4, F84.5, F84.8, F84.9; F88, F89, F90.0, F90.1, F90.2, F90.8, F90.9
**Tic disorders**	F95.0, F95.1, F95.2, F95.8, F95.9, F98.4, F98.5, R25
**Epilepsy and febrile seizures**	G25.3, G40, G41, R56

### Co-variates

BMI was calculated as weight in kilograms (measured by a midwife) divided by the square of body height (self-reported) in square meters. Mode of delivery was recorded in standardized delivery records. Gestational age, birth weight and Apgar scores were registered in the neonatal record. In 94.3% of pregnancies, gestational age was based on ultrasound examination, offered to all women in early second trimester. If data on ultrasound was not available, last menstrual period was used for pregnancy dating. Information on maternal body-mass index (BMI) and parity were collected at the first attendance to antenatal care. Self-reported data on smoking was collected at 32 gestational weeks. Birth weight by gestational age was calculated using the sex-specific Swedish reference curve for normal fetal growth [18]. Appropriate for gestational age (AGA) was defined as the 10th to the 90th percentile of expected birth weight for sex and gestational age. Small for gestational age (SGA) was defined as less than the 10th, and large for gestational age (LGA) was defined as more than the 90th percentile of expected birth weight for gestational age and sex.

### Statistical analysis

Mode of delivery, gestational age, birth weight for gestational age, infant sex, Apgar score and birth year were a priori considered possible confounders or mediators and were considered by stratification, restriction and/or adjustment in the multivariable analyses. Early pregnancy BMI, parity and smoking in gestational week 32 did not affect the relationship between neonatal hypoglycemia and neurological or neurodevelopmental outcome, and thus, these variables were not included in the final regression models. A logistic regression model was used to determine crude odds ratios and 95% confidence intervals. Analyses started with the main outcome and then secondary outcomes were evaluated one by one. Co-variates were first tested separately and, if contributing to the association between exposure and outcome, they were added into a stepwise multivariable regression model. Parity, mode of delivery, gestational age and Apgar score were a priori considered possible effect modifiers on the association between neonatal hypoglycemia and subsequent neurodevelopmental outcome. Effect modification was tested by stratification and insertion of an interaction variable in the regression models. A *P* value < 0.05 was considered statistically significant. For statistical analyses, SAS version 9.4 was used.

## Results

In the study population, 1 500 infants (1.5%) had a hypoglycemia diagnosis at discharge from the postnatal ward. Risk factors associated with a high proportion (≥ 2.0%) of hypoglycemia diagnosis were: being a first-born, maternal BMI ≥ 30, maternal smoking, elective Cesarean delivery, emergency Cesarean delivery, preterm birth < 37 gestational weeks, term birth at 37–38 weeks (as compared to term birth at 39–40 weeks), SGA, LGA, and Apgar score less than 7 at 5 min (Table 2).

**Table 2:** Maternal, delivery and infant characteristics

Characteristics	Hypoglycemia (n = 1 500)	No hypoglycemia (n = 99,560)	Significant difference yes/no*
	Number (%)	Number (%)	
Maternal age (years)			
< 25	158 (11)	9783 (10)	No
25–29	343 (23)	24,162 (24)	No
30–34	542 (36)	37,415 (38)	No
≥ 35	457 (30)	28,200 (28)	No
Parity (including present birth)			
1	917 (61)	44,835 (45)	Yes
2	350 (23)	37,597 (38)	Yes
≥ 3	233 (16)	17,128 (17)	No
Maternal BMI (kg/m^2^)			
< 18.5	40 (3)	2788 (3)	No
18.5–24.9	841 (56)	64 446 (67)	Yes
25–29.9	369 (25)	20,504 (21)	Yes
≥ 30	194 (13)	7933 (8)	Yes
Missing		3945	
Daily smoking in beginning of pregnancy			
Non-smoker	1405 (94)	95,142 (96)	Yes
Smoker	93 (6)	4324 (4)	Yes
Missing		96	
Mode of delivery			
Vaginal non-instrumental	767 (51)	73,656 (74)	Yes
Vaginal instrumental	148 (10)	8327 (8)	Yes
Planned cesarean section	283 (19)	9375 (9)	Yes
Emergency cesarean section	302 (20)	8202 (8)	Yes
Infant sex			
Male	896 (60)	49,730 (50)	Yes
Female	604 (40)	49,830 (50)	Yes
Gestational week			
34	6 (0.4)	22 (0.02)	Yes
35	19 (1)	135 (0.1)	Yes
36	103 (7)	1212 (1)	Yes
37	192 (13)	4224 (4)	Yes
38	299 (20)	14,437 (15)	Yes
39	275 (18)	25,249 (25)	Yes
40	317 (21)	29,222 (29)	Yes
41	190 (13)	18,859 (19)	Yes
≥ 42	92 (6)	6,155 (6)	Yes
Missing		52	
Birth weight for gestational age			
Small for gestational age (SGA)	289 (19)	5386 (5)	Yes
Appropriate for gestational age (AGA)	933 (62)	83,728 (84)	Yes
Large for gestational age (LGA)	278 (19)	10,385 (10)	Yes
Missing		61	
Apgar score at 5 min			
7 or more	1475 (98)	99,168 (100)	Yes
> 7	23 (2)	204 (0.2)	Yes
Missing		190	
Birth year			
2008	179 (12)	9643 (10)	Yes
2009	295 (20)	21,839 (22)	Yes
2010	356 (24)	22,985 (23)	No
2011	303 (20)	22,503 (23)	Yes
2012	367 (24)	22,590 (23)	No

Congenital malformations, inborn errors of metabolism, and maternal diabetes are excluded in the Stockholm-Gotland cohort 2008–2012

*Variable significantly different between hypoglycemia and non-hypoglycemia group in chi2-test (*P* < 0.05)

Infants treated for moderate or transient neonatal hypoglycemia had over 50% higher rates of any neurological or neurodevelopmental outcome at follow-up, and rates of any developmental delay, motor and cognitive developmental delay were at least doubled as compared to normoglycemic infants. Rates of autism spectrum and attention-deficit/hyperactivity disorders, tics and stereotypic behavior, and epileptic and febrile seizures were 30–50% higher among infants with hypoglycemia. In regression analyses, the crude risk of any neurological or neurodevelopmental outcome was increased by approximately 60% in hypoglycemic infants compared to normoglycemic peers. After adjustment for birth weight for gestational age, gestational age, mode of delivery, sex, Apgar score at 5 min, and birth year, the risk remained almost 50% increased. The crude risk of any developmental delay was tripled among hypoglycemic infants and the risk decreased somewhat after adjustment. The risk of motor developmental delay was doubled among hypoglycemic infants in both crude and adjusted analyses. The risk of cognitive developmental delay was almost quadrupled in crude analyses. After adjustment, the risk was almost tripled. Risks for autism spectrum and attention-deficit/hyperactivity disorders, tics and stereotypic behavior, epileptic and febrile seizures were not statistically significant and were omitted from further analyses (Table 3).

**Table 3:** Numbers, rates and risks of adverse neurodevelopmental outcomes

	Total number	Hypoglycemia N = 1500		No hypoglycemia N = 99,560		Logistic regression Reference group = No hypoglycemia			
		Number	Rate/1000	Number	Rate/1000	Crude		Adjusted^a^	
						OR	95% CI	OR	95% CI
Any neurological or neurodevelopmental outcome	3371	77	51	3294	33	1.58	1.25–1.99	1.48	1.17–1.88
Any developmental delay	675	29	19	646	6.5	3.02	2.07–4.40	2.53	1.71–3.73
Motor developmental delay	393	12	8.0	381	3.8	2.10	1.18–3.74	1.91	1.06–3.44
Cognitive developmental delay	314	17	11	297	3.0	3.83	2.34–6.26	2.85	1.70–4.76
Autism spectrum and attention-deficit/hyperactivity disorders	313	7	4.7	306	3.1	1.52	0.72–3.22	1.04	0.48–2.24
Tics and stereotypic behaviour	100	2	1.3	98	1.0	1.36	0.34–5.50	1.43	0.35–5.87
Epileptic seizures and febrile seizures	2456	45	30	2411	24	1.25	0.92–1.68	1.23	0.91–1.67

Congenital malformations, inborn errors of metabolism, and maternal diabetes are excluded

^a^Adjusted for mode of delivery, birth weight for gestational age, gestational age, sex, Apgar score and birth year

Among infants with a hypoglycemia diagnosis, 383 had the diagnosis P70.4A (neonatal hypoglycemia before 6 h of age) and 1 013 had the diagnosis P704.B (neonatal hypoglycemia after 6 h of age). The remainder of hypoglycemic infants (n = 104) were not specified according to timing and these infants were excluded from analyses stratified by timing of hypoglycemia. Infants with early neonatal hypoglycemia had a 2-5 fold increased risk of affected neurodevelopment compared to normoglycemic infants in crude analyses. After adjustments, the specified risks of any neurological or neurodevelopmental outcome, any developmental delay and cognitive developmental delay were doubled to tripled. Infants with neonatal hypoglycemia after 6 h of age had a more than doubled to tripled risk of any developmental delay, motor developmental delay and cognitive developmental delay compared to their normoglycemic peers. In adjusted analyses, the risk of any developmental delay was more than doubled and the risk of cognitive developmental delay was more than doubled (Table 4).

**Table 4:** Risks of adverse neurodevelopmental outcome by early and late neonatal moderate hypoglycemia

	Early hypoglycemia < 6 h N = 383				Late hypoglycemia > 6 h N = 1013			
	Crude		Adjusted^a^		Crude		Adjusted^a^	
	Odds ratio	95% CI	Odds ratio	95% CI	Odds ratio	95% CI	Odds ratio	95% CI
Any neurological or neurodevelopmental outcome	2.22	1.50–3.28	1.94	1.30–2.89	1.33	0.98–1.80	1.29	0.95–1.76
Any developmental delay	4.10	2.18–7.72	3.01	1.57–5.79	2.61	1.61–4.24	2.33	1.42–3.82
Motor developmental delay	2.75	1.02–7.40	2.34	0.86–6.41	2.08	1.03–4.19	1.93	0.95–3.92
Cognitive developmental delay	5.30	2.35–11.98	3.17	1.35–7.43	2.99	1.54–5.82	2.54	1.29–5.01

Congenital malformations, inborn errors of metabolism, and maternal diabetes are excluded

^a^Adjusted for mode of delivery, birth weight for gestational age, gestational age, sex, Apgar score and birth year

In the study population, 5 675 (5.6%) infants were SGA, and 10 688 (10.6%) infants were LGA. Crude risks of any neurological or neurodevelopmental outcome among hypoglycemic infants by birth weight percentiles are shown in Table 5. Because of lack of power in the stratified analyses, adjusted odds ratios did not reach statistical significance in any strata, and thus only crude associations are shown. SGA infants with neonatal hypoglycemia had a doubled risk of any neurological or neurodevelopmental outcome compared to their normoglycemic peers, whereas risks of any/motor/cognitive developmental delay were non-significant. The risks of adverse outcome at follow-up, and especially cognitive developmental delay, were significantly increased among AGA hypoglycemic infants. For LGA hypoglycemic infants, we did not detect any increased risks for the investigated outcomes.

**Table 5:** Risks of adverse neurological outcome by birth weight groups

	Small for gestational age n = 5 675				Appropriate for gestational age n = 84 722				Large for gestational age n = 10 688			
	Number	Rate/1000 live born	OR	95% CI	Number	Rate/1000 live born	OR	95% CI	Number	Rate/1000 live born	OR	95% CI
Any neurological or neurodevelopmental outcome	225	39.6			2815	33.2			331	31.0		
No hypoglycemia	203		1.00	Reference	2769		1.00	Reference	322		1.00	Reference
Hypoglycemia	22		2.10	1.33–3.32	46		1.52	1.13–2.05	9		0.95	0.50–1.79
Any developmental delay	58	10.2			549	6.48			68	6.36		
No hypoglycemia	52		1.00	Reference	529		1.00	Reference	65		1.00	Reference
Hypoglycemia	6		2.18	0.93–5.11	20		3.45	2.20–5.41	3		1.92	0.70–5.29
Motor developmental delay	33	5.81			317	3.74			43	4.02		
No hypoglycemia	30		1.00	Reference	309		1.00	Reference	42		1.00	Reference
Hypoglycemia	3		1.87	0.57–6.17	8		2.34	1.16–4.73	1		1.47	0.35–6.07
Cognitive developmental delay	29	5.11			256	3.02			29	2.71		
No hypoglycemia	26		1.00	Reference	244		1.00	Reference	27		1.00	Reference
Hypoglycemia	3		2.16	0.65–7.15	12		4.46	2.49–8.00	2		2.34	0.55–9.87

Congenital malformations, inborn errors of metabolism, and maternal diabetes are excluded

We did not detect any effect modification from parity, mode of delivery, gestational age, or 5-min Apgar score on the association between neonatal hypoglycemia and neurological diagnoses at follow-up.

## Discussion

In this population-based cohort study including 101 060 infants followed-up until 2-6 years of age, moderate neonatal hypoglycemia was associated to increased risks of later neurological diagnoses. More specifically, the risks were increased for developmental delay and risk estimates were robust through multivariable analyses adjusting for confounding factors.

In publications from the New Zealand CHYLD cohort, high-risk infants (58.9% had neonatal hypoglycemia) were extensively followed-up at 2 (n = 404) and 4.5 years (n = 477) [7, 11]. At 2 years of age, the authors found no increased risk of neurosensory impairment or processing difficulties in toddlers, as measured by validated questionnaires and tests. At 4.5 years, neonatal hypoglycemia was associated with two- to three- fold increased risks of poor executive and visual motor performance. The results of our study are consistent with the CHYLD results as well as previous smaller studies where moderate hypoglycemia has been associated with cerebral white matter injury at 6 weeks postnatal age [3], injury of occipital white matter and in the thalamus [4], lower results on the Bailey motor and mental development scales at 18 months [5] and poor performance on the 4th grade Literacy and Mathematics achievements test [13]. However, these studies have been either underpowered to detect rare outcomes [3–5, 13], have included a very short follow-up [3–5], and/or have been selected from a high-risk population, such as preterm infants [5] or infants with symptoms from the central nervous system [3, 4], which in itself may increase the risk of adverse outcomes. Some of these studies have shown an improvement of the outcome over a short or long follow-up time [3, 4, 19], and one of the studies could only detect a negative effect of moderate hypoglycemia if it lasted for several days [5]. In a study of moderately preterm infants, hypoglycemia was identified as the only neonatal morbidity that could predict an impaired neurodevelopment as reported by parents at 4 years of age [2]. The authors argued for a causal association and suggested intensified monitoring of glucose levels in moderately preterm infants. Two other studies have failed to associate moderate hypoglycemia to impaired neurodevelopmental outcomes [11, 12]. In addition to the 2-year follow-up of the CHYLD study, a psychometric assessment of 38 hypoglycemic preterm infants and an equal number of controls at age 15, found that the groups were similar regarding outcomes [12].

Our results on birth weight for gestational age percentiles were unexpected. The rates of the compiled neurological or neurodevelopmental outcome were of a similar magnitude in SGA and AGA infants, whereas risks of developmental delay were increased in AGA but not in SGA infants. This may be due to chance or insufficient power. It may also be a variant of the Low Birth Weight Paradox, as suggested by Wilcox [20], where low birth weight infants in some high-risk groups (smokers, African Americans, residents of high altitude) paradoxically have better outcomes than their normal weight peers. Moreover, we did not find any significant associations in the LGA group. The study size should rule out power problems at least for the any neurological or neurodevelopmental outcome. An explanation for the lack of association may be that we excluded mothers with diabetes, and thus ended up with a relatively healthy group of LGA infants with an intrinsic low risk of neurological diseases.

In our cohort, we were able to analyze 1500 otherwise healthy cases of moderate neonatal hypoglycemia and to compare them with 99,560 healthy reference infants. This is the largest study on moderate neonatal hypoglycemia so far. The Stockholm-Gotland cohort has provided us with population-based [16], high-quality and complete data on exposure, co-variates and confounders, and we had enough power to make relevant adjustments and stratifications. To our knowledge, no previous studies have been able to exclude children who were later diagnosed with inborn errors of metabolism. Children born with metabolic diseases may have neonatal hypoglycemia as their first isolated symptom, sometimes long before diagnosis, and they also have a higher risk of impaired neurodevelopment. The possibility to exclude metabolic disorders significantly reduces the risk for reversed causality. As the first report known to us, we also had data on the timing of hypoglycemia (early, < 6 h, or late, > 6 h) in 93% of the exposed infants.

There are several limitations to our study; the first is that we have used discharge diagnoses as the exposure. Diagnosis status is a blunt instrument and there may be missed cases of moderate hypoglycemia where the diagnosis was not entered by the doctor at discharge. There may also be an unknown number of infants who were in fact hypoglycemic but went undetected. However, exposure misclassification is not associated with how the outcome was measured, suggesting non-differential misclassification. This bias would thus dilute the effects and not exaggerate them. Second, we did not have data on blood glucose levels. We were able to exclude all infants treated in the neonatal intensive care unit. These were infants with very low blood glucose levels (< 27 mg/dL), with prolonged hypoglycemia or where oral treatment failed. This exclusion provided a study population of otherwise healthy newborns who were at low risk of complications. The exclusions of severe hypoglycemia and infants of mothers with diabetes also resulted in a low percentage of hypoglycemia in the cohort (1,5%). Nevertheless, results should be generalizable to most newborn infants of non-diabetic mothers that we see and treat in the clinical setting. Third, we had a follow-up of two to 6 years of age, which is enough only to detect major adverse neurodevelopmental outcomes. Infants and toddlers in Sweden are routinely screened with standardized developmental tests by a trained pediatric nurse at 2; 4; 6; 8; 10; 12; and 18 months of age and at 3, 4 and 5 years of age. Concern for a delayed development will lead to a referral to a pediatrician or a pediatric neurologist. Almost all Swedish children participate in the screening programme. Due to the large study population, it was not possible to measure neurodevelopment in all included children. Children who develop problems later in life than 2–6 years of age, will be misclassified as healthy in this study. This situation is similar for both the exposed and unexposed group. It would be interesting to see the results of a longer follow-up, perhaps with more detailed data on cognitive development and capacity. Fourth, our study has an observational design that cannot prove a causal relationship between moderate neonatal hypoglycemia and adverse neurodevelopment, and reversed causality cannot be completely ruled out. Fifth, we did not have data on heredity or the occurrence of neuropsychiatric, neurological or neurodevelopmental diagnoses in the parents. For these factors to act as confounders, they would have to be associated to both the exposure and the outcome. This may be the case in some instances, thus introducing a differential misclassification, which in most cases would exaggerate the risk. We do not believe that this is sufficiently common to affect our results to any significant degree. We also lacked data on other socio-economic factors than maternal BMI and smoking habits, which were found to have no influence on the study results. Parent education, housing status and income may have confounder status. Due to lack of data on these variables in the study, we used maternal smoking and BMI as proxys for other life-style factors, but there may still be some residual confounding. Sixth, our outcomes and co-variates were pre-defined based on previous studies and clinical experience. However, we did not perform any statistical test to rule out multiple test errors and there is, as always, a potential for chance findings in our results especially among the smaller groups of specific neurological outcomes and some caution should be taken when interpreting those results. Even though the risk for adverse neurodevelopmental outcome is clearly higher in the hypoglycemic group, the absolute risk of having any neurological or neurodevelopmental outcome is still relatively small (prevalence of 5.1% in the moderately hypoglycemic group versus 3.3% in the normoglycemic group. Nevertheless, the finding of relatively high and consistent risk measures, lack of the above mentioned biases and the support from previous studies imply that part of the association may be causal.

Our study contributes to the dispute on moderate neonatal hypoglycemia and the possibility of adverse long-term outcome, by providing a large population-based study of low-risk infants who showed increased risks of neurological and neurodevelopmental outcomes. Our results support the current treatment practice of blood glucose sampling as an immediate action in symptomatic infants, also in low-risk infants, as well as the routines of extra oral feeding in infants with moderate hypoglycemia and preventive feedings to high-risk infants. Contrary to general belief, we found a significantly elevated risk of developmental delay in infants with moderate neonatal hypoglycemia starting before 6 h of age. In this time span, a decrease of blood glucose is considered to be physiologic. Our findings indicate that hypoglycemia during the first 6 h of age may be associated to a negative impact on the newborn brain and that treatment could be equally important in this early phase.

## Conclusions

We conclude that moderate neonatal hypoglycemia is associated with increased risks of impaired neurodevelopment in pre-school children. Our data do not support the notion that moderate neonatal hypoglycemia is a harmless physiologic state, or that early hypoglycemia is less damaging than later onset hypoglycemia. Clinical guidelines with screening of symptomatic and high-risk infants should be followed and immediate treatment of hypoglycemia should be provided.

## Abbreviations

ICD-10: International classification of diseases 10
BMI: Body-mass index
AGA: Appropriate for gestational age
SGA: Small for gestational age
LGA: Large for gestational age
OR: Odds ratio
CI: Confidence interval
